# High Throughput Studies of Cell Migration in 3D Microtissues Fabricated by a Droplet Microfluidic Chip

**DOI:** 10.3390/mi7050084

**Published:** 2016-05-05

**Authors:** Xiangchen Che, Jacob Nuhn, Ian Schneider, Long Que

**Affiliations:** 1 Department of Electrical and Computer Engineering, Iowa State University; Ames, IA 50011, USA; che@iastate.edu; 2 Department of Chemical and Biological Engineering, Iowa State University; Ames, IA 50011, USA; januhn@iastate.edu; 3 Department of Genetics, Development and Cell Biology, Iowa State University; Ames, IA 50011, USA

**Keywords:** cell motility, autocrine, paracrine, 3D micro-sized tissue, microfluidic droplet device

## Abstract

Arrayed three-dimensional (3D) micro-sized tissues with encapsulated cells (microtissues) have been fabricated by a droplet microfluidic chip. The extracellular matrix (ECM) is a polymerized collagen network. One or multiple breast cancer cells were embedded within the microtissues, which were stored in arrayed microchambers on the same chip without ECM droplet shrinkage over 48 h. The migration trajectory of the cells was recorded by optical microscopy. The migration speed was calculated in the range of 3–6 µm/h. Interestingly, cells in devices filled with a continuous collagen network migrated faster than those where only droplets were arrayed in the chambers. This is likely due to differences in the length scales of the ECM network, as cells embedded in thin collagen slabs also migrate slower than those in thick collagen slabs. In addition to migration, this technical platform can be potentially used to study cancer cell-stromal cell interactions and ECM remodeling in 3D tumor-mimicking environments.

## 1. Introduction

It is very difficult to uncover how cells respond to the extracellular matrix (ECM) and how cells communicate using traditional cell culture systems. Traditional cell culture systems offer only two-dimensional (2D) substrates and lack the ability to isolate single cells or groups of cells [1,2].

An ideal platform for high throughput studies of cell-ECM interactions and cell-cell communication must have the following characteristics: (1) The platform is capable of realizing the encapsulation of cells in an ECM similar to that in the body, and the ECM should be three-dimensional (3D). (2) The platform can realize the isolation of single cells or groups of cells in order to control the cell-cell communication. This implies confining cells by providing barriers between the cell environment and the surroundings. (3) The platform must allow one to build microenvironments that are sufficiently small such as microtissues. Cells often communicate through the secretion of soluble molecules, so volumes between 10- to 1000-fold larger than the cell are appropriate to ensure that the secreted molecule concentration is sufficiently high. (4) Lastly, the platform is capable of rapidly generating a large number of cell-encapsulated microtissues in parallel in a cost-effective manner for high throughput studies.

## 2. Design, Operational Principle and Fabrication of the Droplet Microfluidic Device

**Figure 1:**
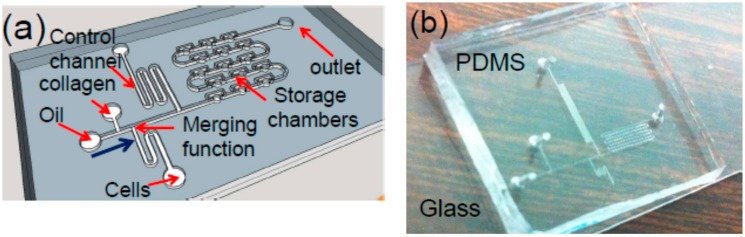
(**a**) Sketch of the droplet microfluidic chip for generating 3D microtissues (*not to scale*): Each storage chamber (a cylinder with a radius of 60 µm and height of 50 µm) has one 3D microtissue containing single or multiple cells; (**b**) Photo of a fabricated chip with 75 storage chambers.

Toward this goal, a droplet microfluidic chip for generating arrayed cell-encapsulated microtissues has been developed [3,4,5]. A schematic of a chip is given in Figure 1a. Filtered silicone oil is used as the continuous flow phase and the carrier fluid. Along the flowing direction of the fluids, as illustrated in Figure 1a, this device consists of a T-shape droplet generator, a liquid-droplet merger, a serpentine control channel (c-channel), and the droplet storage chambers (chambers). The droplet generator forms cell-laden collagen droplets. The c-channel is designed to prevent any air bubbles or non-uniform droplets from entering and occupying the chambers at the beginning of the operation of the device [6,7]. Once the uniform droplet generation is established, the c-channel is closed, and the outlet of the chambers is open. As a result, the droplets will flow toward the chambers, thereby entering and occupying them one by one. It should be noted that while this type of chip has been used for other applications [6], it is for the first time to be used to generate 3D microtissues and study the migration of the cells. 

A large scale of arrayed 3D microtissues, formed by polymerized collagen and cells, can be manufactured and stored in microchambers on the chip. To the best of our knowledge, this is the first demonstration of the fabrication of 3D microtissues using a droplet microfluidic chip to study cell migration in 3D microenvironments for an extended period of time.

The chip is fabricated using a soft lithography process [6,7]. Briefly, a 50-µm-thick SU-8 mold of the device is formed on a silicon substrate using conventional optical lithography. Polydimethylsiloxane (PDMS) is then casted on the mold, followed by 1.5 h of curing at the temperature of 65 °C. Finally, the PDMS microfluidic layer is peeled off from the mold, and then is bonded with a glass substrate after oxygen plasma treatment for 10 s. The input and output holes are made in the PDMS layer for the delivery of the samples to the chip, followed by assembling input and output tubing (Upchurch Scientific, Inc., Oak Harbor, DC, USA), and being connected with syringes controlled by several syringe pumps (KD Scientific, Inc., Holliston, MA, USA). A photo of a fabricated chip is shown in Figure 1b.

## 3. Materials and Methods

Breast cancer MDA-MB-231 cells were subcultured in Dulbecco’s modified Eagle’s medium with 10% fetal bovine serum, 2% Glutamax, and 1% penicillin/streptomycin. Imaging media was the same except it lacked phenol red and was supplemented with 12 mM HEPES. On the day the chambers were loaded, cells were trypsinized and suspended in 2 mg/mL collagen solution (rat tail-CORNING-354249) neutralized with imaging media at a cell density of 2 × 10^6^ cells/mL. Chips were either loaded with a continuous collagen phase or with droplets in the storage chambers. Cells were allowed to spread over 24 h. Phase contrast images were then taken every 0.5–2 min over 4–8 h. Slabs of collagen were generated between two microscope slides with 60 µm (thin) or 360 µm (thick) spacers. Cells were prepared and imaged in the same way as for the devices with the exception that 3 × 10^5^ cells/mL were used.

In order to mitigate or even eliminate the droplet shrinking issue due to evaporation, the fabricated chip was firstly soaked in PBS buffer solution (pH-7.4) in incubator (FISHER SCIENTIFIC-ISOTEMP 3530) overnight before use to ensure that PDMS was saturated with PBS. Silicone oil (SIGMA-ALDRICH) was used as the fluid carrier. Harvard syringe pump (70–4500) was connected with syringes for flowing the oil and the collagen/cells. In the experiments, the cell loading in the collagen droplets was based on Poisson distribution without any attempt to control the loading process. In addition, no surfactant was used to facilitate the droplet stability. During the collagen droplet generation and storage process, the collagen flowing input tube and syringe were submerged into a cold water tank (0~2 °C) to avoid fast polymerization since the polymerization rate is highly depended on temperature. After the droplets were stored in the chambers, the device was flipped over every minute within 10 min until the collagen was fully polymerized in the storage chamber, and to make sure the cells were in the middle of the storage chamber (along the *z*-axis), thereby ensuring the cells to stay in the 3D-matrix. For the experiments, the droplet microtissues remain surrounded by silicone oil. Experiments on the cell behaviors after the oil is replaced by cell culture media are in progress.

Confocal reflectance microscope (LEICA LAS-AF, Weltzlar, Germany) was used to image the 3D-matrix system. Standard incubator (FISHER SCINTIFIC-ISOTEMP 3530, FISHER SCINTIFIC, Waltham, MA, USA) was used to incubate the chip overnight in order to make cells accommodate to 3D-matrix system for cells’ optimum behavior. OLYMPUS IX73 (OLYMPUS, Tokyo, Japan) with camera DP73 (OLYMPUS, Tokyo, Japan) was used to track the cell migration.

During the cell tracking process, the chip was submerged into a glass petri dish filled with PBS buffer at 37 °C to prevent drying problem. A heating stage (HARVARD APPARATUS-c-11842, HARVARD APPARATUS, Holliston, MA, USA) was applied to supply continuous heat. Finally, image J (National Institutes of Health, Bethesda, MD, USA) with a cell tracker model was used to track and plot the cell migration diagram. Experiments found that oxygen depletion was not a problem, even in our relatively small microtissues with PDMS and media above. Cell death did not occur over the period of about two days in the chamber, particularly if it was kept under proper pH buffering and temperature conditions. The oxygen consumption rate (OCR) for cancer cells is no higher than 30 pmol·s^−1^·10^−6^·cells [8]. The volume of each microtissue is ~6.0 × 10^−10^ L and no more than 10 cells occupy a microtissue. Consequently, the OCR for one microtissue is 500 nM/s. If no oxygen transfer occurs, it would take over a day for the cells in each microtissue to decrease the oxygen concentration from 260 µM, the saturated level of media in equilibrium with air in the incubator, to 200 µM, a value still well above hypoxic conditions. However, there is oxygen transport across the liquid and PDMS, and the transport is governed by the following equation at steady state: OCR = (*D*/*h*)*A*(C* − *C*), where OCR is the oxygen consumption rate (0.3 fmol/s), *D* is the diffusion coefficient of oxygen in PDMS or water (3 × 10^−5^ cm^2^/s) [9], *A* is the cross-sectional area of each microtissue (1.2 × 10^−4^ cm^2^), C* is the equilibrium concentration of oxygen in fluid (260 µM), *C* is the local oxygen concentration around the cells and *h* is the height of the PDMS and fluid above the microtissue. At a height of 0.8 cm, the steady-state oxygen concentration is about 200 µM. While there is little information on whether cell function is altered at this concentration, it is well above that which is considered hypoxic (<6 µM). Furthermore, because media is initially at an equilibrium concentration of 260 µM oxygen, it takes time for the oxygen concentration to reach this steady state. At the time that experiments are conducted, the oxygen level is 200–210 µM. Consequently, the 0.8 cm of PDMS and media is thin enough to support the relatively low rate of oxygen consumption within the microtissues. 

## 4. Results and Discussion

**Figure 2:**
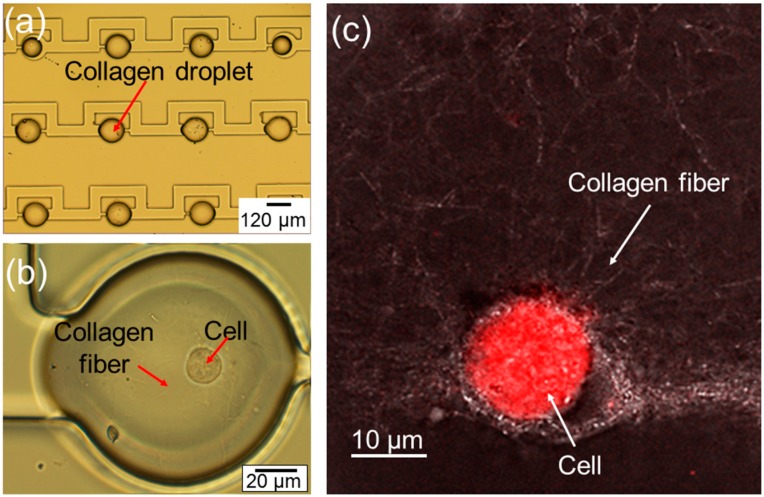
(**a**) Photo of arrayed microtissues stored in storage chambers; (**b**) close-up of one microtissue containing one cell; (**c**) confocal image of one cell inside polymerized collagen fiber, forming a microtissue.

The optical image of the fabricated arrayed microtissues inside the storage chambers is given in Figure 2a. Following the procedure described in Section 2, it has been demonstrated that the uniform microtissues can be formed and stored in the storage chambers on the chip routinely. However, it should be emphasized that care should be taken to avoid the polymerization of the collagen in the flowing channels on the chip; otherwise, the storage chambers cannot be occupied by microtissues properly. In Figure 2b, a close-up optical image of a droplet shows a cell inside a polymerized collagen fiber. In order to show the collagen fiber more clearly, a confocal image in Figure 2c has been taken on the droplet, showing one cell embedded in the polymerized collagen fiber.

**Figure 3:**
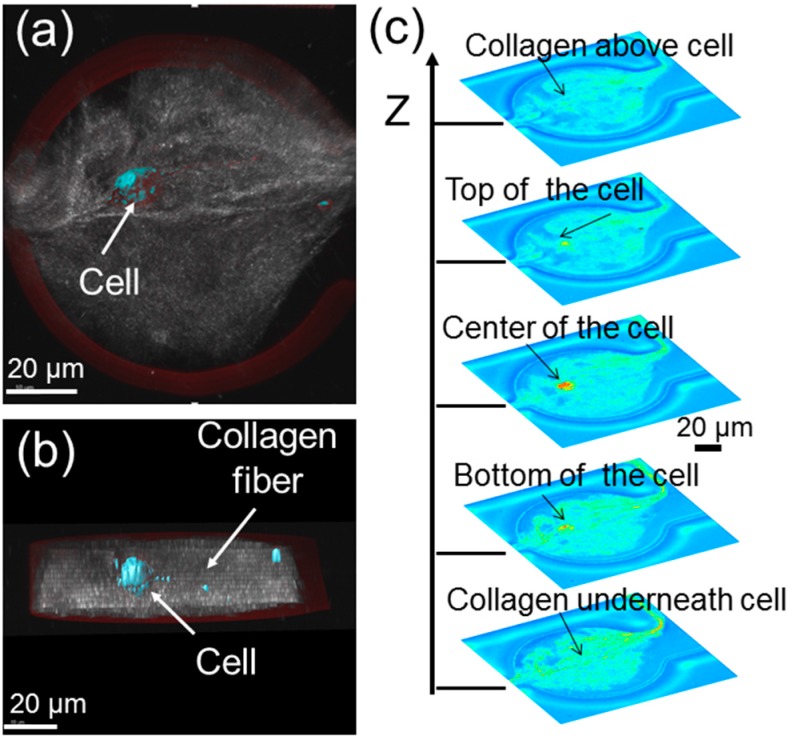
Confocal images showing one cell inside a 3D microtissue in a storage chamber: (**a**) topside view; (**b**) cross-section view; (**c**) stacked confocal images of a microtissue showing one cell inside a 3D microtissue.

In order to confirm that the cell is indeed surrounded by a 3D extracellular matrix (ECM), which is made up of polymerized collagens, some confocal images of the microtissues have been taken. A topside view, cross-section view and the stacked images from the bottom to the top of a microtissue are obtained in Figure 3. Given that the nominal height of the fabricated storage chambers is ~50 µm, the cell is roughly ~20 µm above the bottom of the microtissue and ~20 µm below the top of the microtissue. Basically, the cell is embedded inside the collagen fibers. Note that the gap of the cell from the top and bottom of the microtissue can be readily increased by increasing the height of the storage chambers.

It has been found that as long as the silicone oil does not directly contact the cells, it will not affect cell viability. In the experiments, only the cells embedded within the polymerized collagen have been studied. These cells are not directly exposed to oil. The total time for the cells inside the polymerized collagen for the experiments was up to 32 h, and no clear effect on cell viability was observed during this time period, suggesting that the oil does not diffuse into the microtissue droplets.

It has also been observed that the polymer gel structure has some differences at the interior *versus* the edges of the microtissue droplets. Interactions with surfaces could potentially nucleate collagen fiber assembly or simply act as an adherent surface for collagen fibers. The typical time for the polymerization of the collagen is ~15 min at room temperature, similar to that for collagen polymerization on a glass cover slip. 

**Figure 4:**
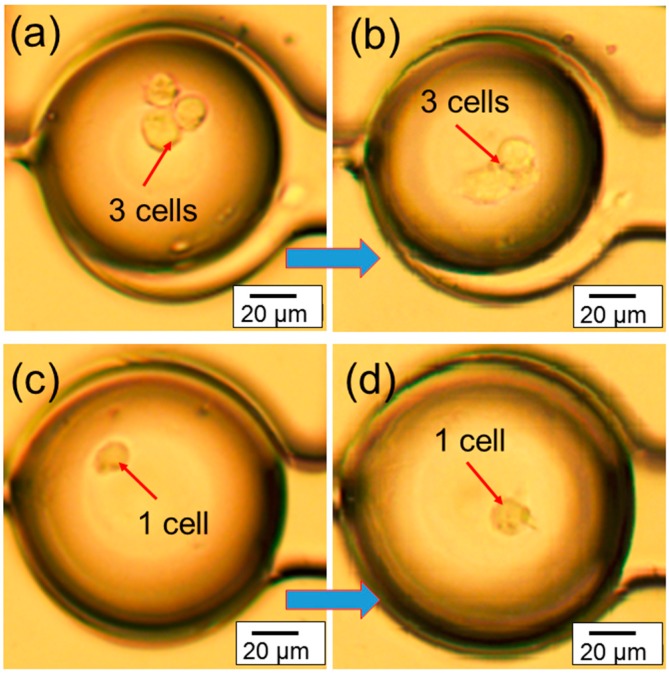
Representative optical images showing (**a**,**b**) the migration of three cells inside 3D microtissue during a 7 h period at 37 °C; (**c**,**d**) the migration of one cell inside 3D microtissue during a 7 h period at 37 °C.

The real-time migration videos (in the supplementary) of the cells inside microtissue have been recorded using an optical microscope. The representative images in Figure 4a,b shows the migration of three congregated cells inside microtissue in a 7 h period of time, while the representative images in Figure 4c,d shows the migration of one cell inside a microtissue during the same period of time. These experiments demonstrate that the chip can provide a platform to study the migration of one single cell or multiple cells in a microtissue environment. In addition, since the cells are confined in a small volume (~600 pL), the communication among them may be easily studied. 

**Figure 5:**
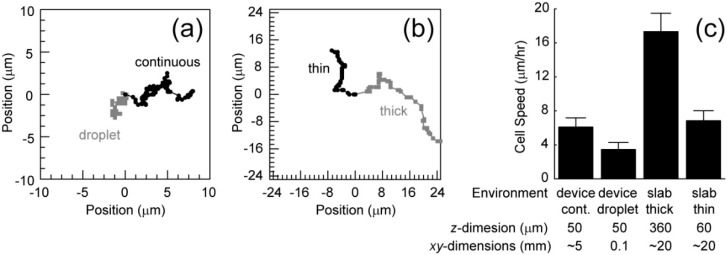
Representative trajectories of cells embedded in collagen (2 mg/mL) in the chip (**a**) and embedded in a collagen (2 mg/mL) slab between two coverslips (thick: grey, thin: black); (**b**) The chip is either filled with a continuous polymerized collagen network (grey) or droplets of collagen within the chambers (black); (**c**) Average cell speed under the different conditions as well as the length scales associated with each condition. Error bars are 95% confidence intervals.

Based on the recorded videos (in the supplementary), the cell migration speed has been calculated under two conditions. The first condition includes chips that are filled with cells embedded in collagen, generating a continuous collagen network. This increases the volume of the environment, decreasing the opportunity for the depletion of nutrients or accumulation of waste. Also, cells in different chambers may communicate. The second condition includes devices that only contain cells embedded in collagen in droplets within the chambers (Figure 4). These droplets have relatively small volumes and cells in a particular droplet cannot communicate with cells in other droplets. These conditions were compared to cell migration in thin (60 µm) and thick (360 µm) slabs of collagen. Representative migration trajectories are shown in Figure 5a,b. Cells in the continuous collagen gels migrate similarly to those in the thin collagen slabs and slower than those in the thick collagen slabs (Figure 5c). Cells in droplets migrated much slower than any other condition (Figure 5c).

It is interesting that the chip filled with a continuous collagen network and a thin slab results in similar migration rates. Collagen stiffness is known to alter migration speeds and the observed stiffness of flexible networks changes close to stiff interfaces, a so-called wall effect. The similar *z*-dimension length scales between these conditions likely generate the similar migration speeds. Consequently, thicker polymerized collagen networks in the chips are likely needed to observe faster migration. Finally, the droplet *xy*-dimensions length scales are much smaller than the other conditions suggesting that either (1) cells require communication between chambers or (2) small volumes in this first generation chip inhibit migration. The second generation chips with storage chambers that are both thicker and larger will allow us to eliminate the wall effects and focus on cell-cell communication within and between chambers that governs cell migration.

## 5. Conclusions 

Using microfluidic droplet chips, arrayed 3D microtissues were fabricated successfully. One or multiple breast cancer cells were embedded within the microtissues. The migration trajectory of the cells was recorded and analyzed. The migration speed inside 3D microtissues was in the range of 3–6 µm/h. It was found that cells in chips filled with a continuous collagen network migrated faster than those where only isolated droplets were arrayed in the chambers. Besides being used for studying the cell migration, this technical platform can be also potentially useful for studying cancer cell-stromal cell interactions and ECM remodeling in 3D tumor-mimicking environments. 

## Supplementary Materials

The following are available online at http://www.mdpi.com/2072-666X/7/5/84/s1. Video S1: One cell in a microtissue, Video S2: Three cells in a microtissue.
